# Entrainment of circadian rhythms of locomotor activity by ambient temperature cycles in the dromedary camel

**DOI:** 10.1038/s41598-020-76535-y

**Published:** 2020-11-11

**Authors:** Hicham Farsi, Mohamed R. Achaâban, Mohammed Piro, Béatrice Bothorel, Mohammed Ouassat, Etienne Challet, Paul Pévet, Khalid El Allali

**Affiliations:** 1 Comparative Anatomy Unit, Department of Biological and Pharmacological Veterinary Sciences, Hassan II Agronomy and Veterinary Medicine Institute, Rabat-Instituts, BP: 6202, 10101 Rabat, Morocco; 2 Medicine and Surgical Unit of Domestic Animals, Department of Medicine, Surgery and Reproduction, Hassan II Agronomy and Veterinary Medicine Institute, Rabat, Morocco; 3 Institute of Cellular and Integrative Neurosciences, CNRS and University of Strasbourg, Strasbourg, France

**Keywords:** Ecology, Neuroscience, Physiology

## Abstract

In the dromedary camel, a well-adapted desert mammal, daily ambient temperature (T_a_)-cycles have been shown to synchronize the central circadian clock. Such entrainment has been demonstrated by examining two circadian outputs, body temperature and melatonin rhythms. Locomotor activity (LA), another circadian output not yet investigated in the camel, may provide further information on such specific entrainment. To verify if daily LA is an endogenous rhythm and whether the desert T_a_-cycle can entrain it, six dromedaries were first kept under total darkness and constant-T_a_. Results showed that the LA rhythm free runs with a period of 24.8–24.9 h. After having verified that the light–dark cycle synchronizes LA, camels were subjected to a T_a_-cycle with warmer temperatures during subjective days and cooler temperatures during subjective nights. Results showed that the free-running LA rhythm was entrained by the T_a_-cycle with a period of exactly 24.0 h, while a 12 h T_a_-cycle phase advance induced an inversion of the LA rhythm and advanced the acrophase by 9 h. Similarly, activity onset and offset were significantly advanced. All together, these results demonstrate that the Ta-cycle is a strong zeitgeber, able to entrain the camel LA rhythm, hence corroborating previous results concerning the T_a_ non-photic synchronization of the circadian master clock.

## Introduction

In order to survive, mammals have evolved several adaptation strategies to cope with ecological pressures of their biotope. In order to escape or enter a synchronous state with biotic and abiotic environmental factors, animals regulate their 24-h general activity patterns to be nocturnal, diurnal, crepuscular or even cathemeral^1–4^. Thus, predation avoidance, availability of food and partners for reproduction, temperature and many other factors strongly modulate these time-partitioning strategies^2,5–9^. Defining the diel time partitioning of activity in a species and its entrainment by environmental cues requires tracking animal movements and clear experimental demonstrations. This is further complicated when we consider species living in harsh environments like deserts. In such biotopes, components like heat exposure, dehydration and food scarcity determine the survival of these animals and modulate their general activity, rendering its exact pattern hard to establish. The dromedary camel is a diurnal animal^10,11^ which is well-adapted to the hostile desert environment. It’s physiological processes of adaptation culminate in water economy. Among these mechanisms, adaptive heterothermia^12,13^ is certainly one of the most interesting phenomena. It is an exceptional thermoregulatory feature displayed by camels exposed to heat stress and dehydration in order to minimize water expenditure. Under such thermal regulation, ambient temperature (T_a_) modulates the body temperature (T_b_), as is the case in ectotherms (reptiles and amphibians), inducing daily fluctuations of T_b_ over a range of 6–8 °C. The desert T_a_ cycle has been shown to modulate another feature of camel adaptive physiology. Indeed, it has been demonstrated that the daily variation of T_a_ in the dromedary camel, like the LD cycle, is able to synchronize the central circadian clock by shifting two of its outputs, the rhythms of T_b_ and melatonin (Mel)^14^, the first clear demonstration of such temperature dependence in a mammal. This suggests that other circadian rhythms such as locomotor activity (LA) could also be entrained by desert T_a_ cycles. In the present study LA rhythms in dromedary camels under specific indoor experimental conditions have been examined to verify if this rhythm is driven by the circadian clock and whether it is synchronized by light–dark (LD) and T_a_ cycles.

## Results

### Dromedary camels exhibit a circadian rhythm of locomotor activity

**Figure 1:**
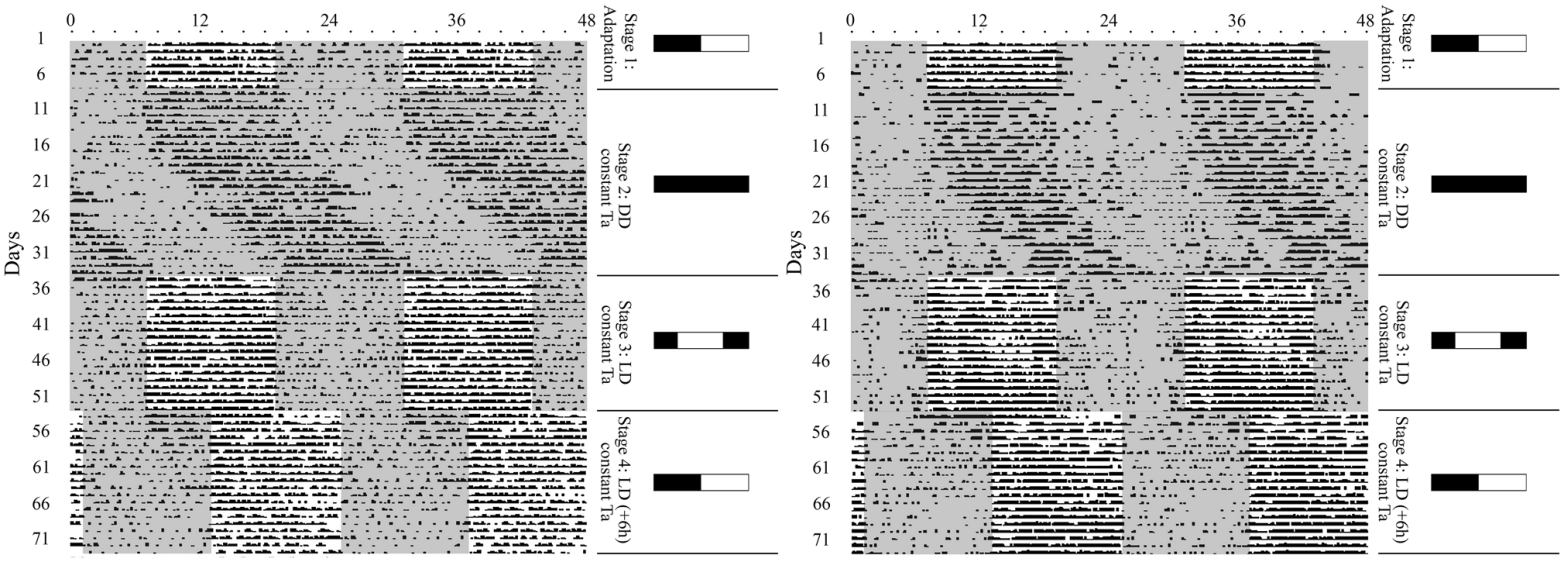
Double plotted actogram of locomotor activity rhythm in two representative dromedary camels during 73 days of experiment 1. Each line corresponds to 24 h of activity starting at 00:00 h and ending at 24:00 h. Black points and lines denote the existence of a locomotor activity rhythm: scores of 1–5. On-line vacuum corresponds to the absence of activity represented by score 0. The different LD-cycle regimes are presented at the right side of the figure. The gray and white areas within actograms represent respectively the dark and the light phases of the LD cycle phases (stages 1, 3 and 4), while the long gray area (Stage 2) denotes constant darkness (DD).

**Figure 2:**
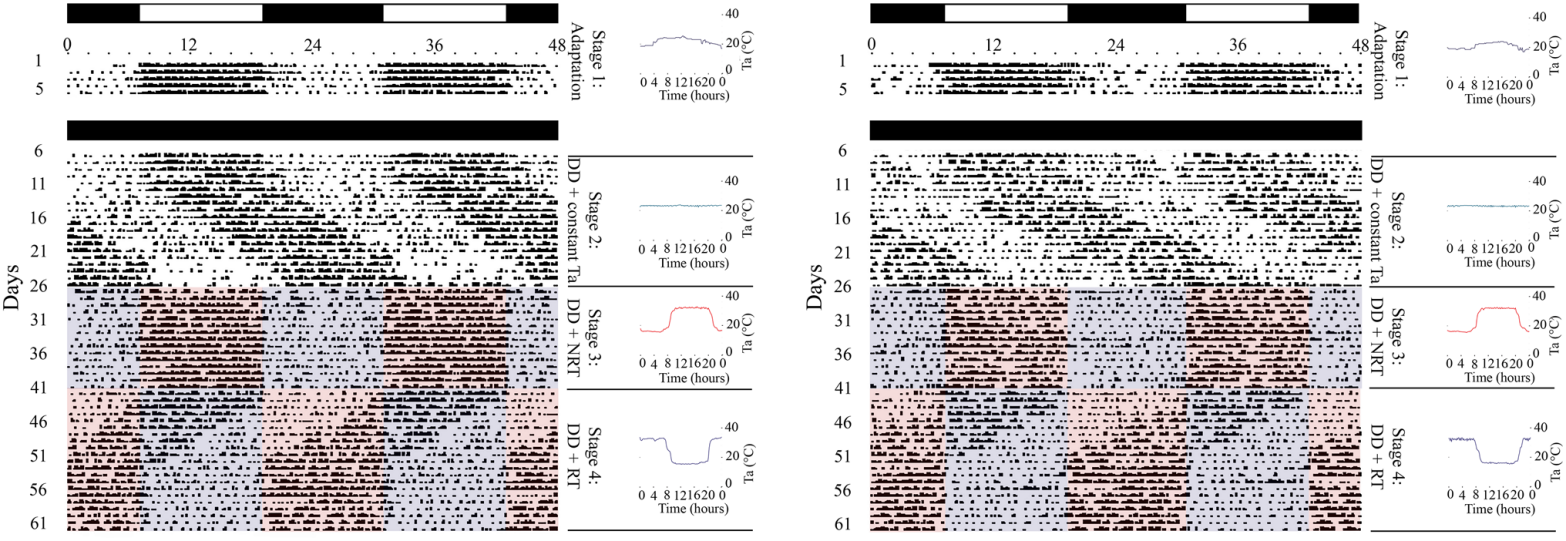
Double plotted actogram of locomotor activity rhythm in two representative dromedary camels during 61 days of experiment 2. Each line corresponds to 24 h of activity starting at 00:00 h and ending at 24:00 h. Black dots and lines denote the existence of a locomotor activity rhythm: scores of 1–5. On-line vacuum corresponds to the absence of activity represented by score 0. The upper black and white bars represent the durations of the LD cycle phases (stage 1); while the long black bar denotes constant darkness (DD) (stages 2–4). The different T_a_ cycle regimes of stages 1 to 4 are presented at the right side as 24 h daily cycle. Warmer and cooler periods of T_a_ cycles are respectively shown within actograms as red and blue areas.

Under a cyclical environment of LD and an uncontrolled T_a_ cycle (stages 1 of both experiments 1 and 2), camels’ LA shows a clear daily rhythm with a period of exactly 24.0 h (*P* ≤ 0.05) and a high robustness level of 37 to 42% (Figs. 1, 2, Table 1). The activity started early in the photophase and then reached its peak in the middle of the day when acrophase has been shown to occur, around 12:50 h. Thereafter, activity dropped and almost disappeared during the scotophase. Actograms confirm that the high levels of activity, corresponding to scores of 2, 3 and 4, coincide within the light phase/high T_a_, while the lack of activity (scores of 0 and 1) is limited to the dark phase/low T_a_.

**Table 1:** Comparison of the LA rhythm parameters (mean ± SEM) between different stages of experiments 1 and 2.

	Period	Acrophase	Mesor	Amplitude	Robustness (%)
**Experiment 1**					
Stage 1	24.0 ± 0.02 h	12:52 ± 0.14 h	1.70 ± 0.05	1.43 ± 0.06	42.9 ± 1.50
Stage 2	24.8 ± 0.07 h	23:18 ± 0.70 h*	0.90 ± 0.09	0.75 ± 0.03	17.3 ± 1.14
Stage 3	24.0 ± 0.02 h	12:50 ± 0.09 h	1.71 ± 0.04	1.41 ± 0.01	39.7 ± 2.10
Stage 4	24.0 ± 0.05 h	17:44 ± 0.19 h	1.67 ± 0.05	1.47 ± 0.06	40.1 ± 1.50
**Experiment 2**					
Stage 1	24.0 ± 0.02 h	12:55 ± 0.10 h	1.8 ± 0.09	1.50 ± 0.06	37.2 ± 1.30
Stage 2	24.9 ± 0.03 h	04:39 ± 1.84 h*	0.94 ± 0.04	0.94 ± 0.06	16.6 ± 0.61
Stage 3	24.0 ± 0.03 h	12:19 ± 0.31 h	1.53 ± 0.09	1.01 ± 0.04	29.6 ± 0.75
Stage 4	24.0 ± 0.04 h	03:19 ± 0.23 h	1.41 ± 0.04	1.00 ± 0.03	25.0 ± 0.90

*Acrophases under constant conditions were calculated for the last day of stages 2.

During stages 2 (experiment 1 and 2), the LA rhythm persisted in the absence of any temporal cue. Indeed, compared to the daily LA rhythm (24.0 h period) first shown under cyclic indoor conditions of stage 1, camels transferred to constant conditions of stages 2 displayed a circadian rhythm of LA with a period completely different from 24.0 h, respectively of 24.8 ± 0.07 h and 24.9 ± 0.03 h for experiments 1 and 2. The one-way ANOVA confirms the existence of very significant differences (*P* ≤ 0.001) between the circadian period and the period calculated for stages 1. Moreover, a classical free-running diminution in rhythm regularity was noticed in stages 2. This was underlined by the drop (*P* ≤ 0.05) in robustness values to a range of 16 to 17% (Table 1). Likewise, the mean acrophase of all camels calculated for the last day of stages 2 showed a shift by almost 10 to 16 h to take place at 23:18 ± 0.70 h and 04:39 ± 1.84 h, respectively, for experiments 1 and 2. Visual inspection of the actograms (Figs. 1, 2) shows a daily drift (phase delay) of LA, confirming its free-running state. As highlighted in Table 1, both mesor and amplitude also underwent significant changes. These results, together with the persistence of rhythmicity under constant conditions, indicate clearly the existence of a circadian clock in the dromedary camel that drives the LA rhythm.

### Light–dark cycle entrainment

When camels were subjected to a cyclic environment with 12L:12D cycle (stage 3 of experiment 1), LA rhythm became more regular and perfectly synchronized with the LD cycle (Fig. 1). This was highlighted by an increase of robustness which reached 39.7 ± 2.1% (Table 1). Likewise, the circadian period (24.8 h) of the former free-running conditions (Stage 2) was shortened to become exactly 24.0 h, equal to the LD cycle period. Significant differences (*P* ≤ 0.05) occurred regarding these changes of the period through stages 1, 2 and 3.

Actograms (Fig. 2) show that the LD cycle induced a daily progressive phase advance in activity. It required 2 to 3 days to reach a perfect resynchronization with the new light regime. Meanwhile, the acrophase was significantly (*P* ≤ 0.05) advanced by almost 5 h to occur at 12:50 ± 0.09 h instead of 16:45 ± 0.53 h, recorded in the previous stage. The applied LD cycles also induced an increase in the mesor and amplitude (Table 1). All these results clearly indicate that the exposure of camel to an LD cycle following DD conditions results in the synchronization of LA rhythms.

A phase delay of 6-h in the light regime during stage 4 lead to a phase delay in the LA rhythm, but took a few days to occur (Fig. 1). This shift in activity is corroborated by the Cosinor regression showing a significant (*P* ≤ 0.05) delay in acrophase by almost 6 h, the acrophase occurred at 17:44 ± 0.19 h instead of 12:50 ± 0.09, as observed in the previous stage. The other parameters remained unchanged (Table 1). These findings illustrate that LA rhythm in the dromedary camel is circadian and entrained by the light–dark cycle.

### Ambient temperature cycle entrainment

The circadian nature of LA rhythm and its entrainment by LD cycle having been established, the next step was then to verify whether T_a_ cycle is a zeitgeber capable of entraining this rhythm in the camel. When animals were placed under a 24-h T_a_ cycle environment (10-h of warmer temperature and 9-h of cooler temperature) (NRT, Stage 3, experiment 2) with heating during the subjective day and cooling during the subjective night, a robust rhythm of activity was recorded (Fig. 2, Supplementary Fig. S1) with a period of exactly 24.0 h. This period was significantly (*P ≤ *0.05) different from that of the previous stage (constant conditions, τ = 24.9 ± 0.03 h). Furthermore, an improvement in rhythm regularity was noticed (Fig. 2), with an increased robustness reaching 29.6 ± 0.75% (Table 1). These changes suggest that the applied Ta cycle imposes its own period and shape to the LA rhythm. Indeed, actograms showed that high activity levels were synchronized to the warmer period of T_a_ cycle, while inactivity coincides with the cooler period (Fig. 2). The acrophases occurred at 12:19 ± 0.31 h (Table 1).

**Figure 3:**
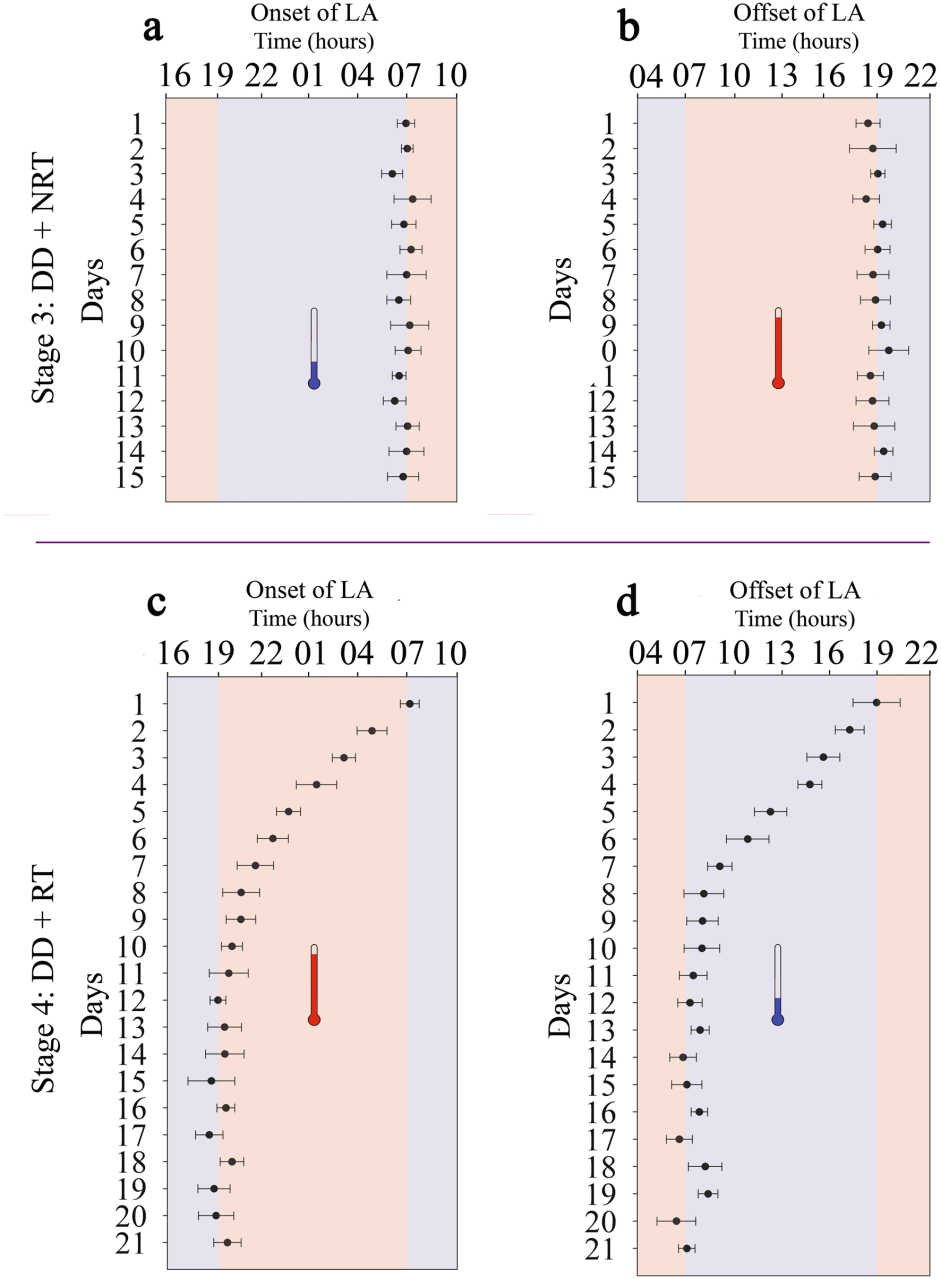
Shift in onset and offset of the LA rhythm (dots with error bars: Means ± SEM) by a phase advance of T_a_ cycle in DD conditions of experiment 2 (camels, n = 6). (**a**,**b**) represent, respectively, the means of onsets and offsets during stage 3 (DD + NRT) in which camels were first maintained under an artificial daily T_a_ cycle with a peak of heat during the subjective day. (**c**,**d**) represent, respectively, the means of onsets and offsets during stage 4 (DD + RT) in which camels were placed under a 12-h advanced T_a_ cycle with a peak of heat during the subjective night. Blue (with blue thermometer symbol) and red (with red thermometer symbol) areas indicate the cold and heat periods of each T_a_ cycle.

The reversal of the T_a_ cycle (RT, 12-h phase advance) in stage 4 (experiment 2) resulted in a significant (*P* ≤ 0.05) total inversion (12-h phase advance) of the LA rhythm (Fig. 2, Supplementary Fig. S1). This phase shifting occurred within a few days, requiring 9.03 ± 1.01 days to be fully synchronized with the new T_a_ regime. The peak of activity switched to occur during the subjective night, which corresponded to the warmer period of the new applied T_a_ cycle. ANOVA analysis showed a significant (*P* ≤ 0.05) advance in the acrophase of LA, which occurred at 03:19 ± 0.23 h (Table 1). As for the acrophase, the activity onset and offset of both stages 3 and 4 confirmed the existence of a phase relationship with the T_a_ cycles. A significant phase advance (*P* ≤ 0.05) of both onsets and offsets were seen when T_a_ was changed from the NRT cycle (stage 3) to a RT cycle (stage 4) (Fig. 3). Results showed that the activity onsets and offsets coincided perfectly with the cold-hot and hot–cold transitions respectively, regardless of the applied T_a_ cycle (Fig. 3). All together, these results clearly demonstrate that the T_a_ cycle is a strong zeitgeber, able to entrain the LA rhythm in the dromedary camel.

## Discussion

In the present work, the circadian nature of LA rhythm and its entrainment by the LD and T_a_ cycles in dromedary camel have been demonstrated unambiguously. Other studies have demonstrated the existence of a circadian clock driving the LA rhythm in some domestic mammals including goat^15^, ram^16^, cat^17^ and rabbit^18^. Such experimental demonstrations are rather well characterized and numerous in laboratory and wild rodents, marsupials, bats and non-human primates^19–25^. Furthermore, the LD cycle entrainment of the LA circadian rhythm has been also well established in several mammalian species including rat^24,26^, mice^27^, Mahali mole rat^28^, rabbit^18^, cat^17^, desert hedgehog^24^ and goat^29,30^. The LD cycle was shown to be the most powerful zeitgeber for the central circadian clock located in the suprachiasmatic nucleus (SCN) of the hypothalamus (for review see^31,32^). Neuroanatomical pathways and mechanisms underlying such photic entrainment are well documented^33–35^. Likewise, the molecular machinery of the central clock is now well established^36–41^. By contrast, much less is known about the neuronal process of non-photic entrainment of the circadian clock. Our results in experiment 2 showed that, in the absence of photic entrainment (DD conditions), a 24.0 h artificial T_a_ cycle with a warmer period during the subjective day and cooler period during the subjective night was able to entrain the free running rhythm of LA of camels. A 12-h phase advance in the daily T_a_ cycle with the warmer period changed to the subjective night and cooler period to the subjective day induced a complete shift (almost 12 h phase advance) in LA rhythm. Whatever the applied T_a_ cycle, the maximum of camel activity always coincided with the warmer period. This corroborates previous observations made in this species under natural conditions which characterized its diurnality, acrophase of LA occurring during daytime^10,11^.

All these results confirm that, in the absence of a photic signal, T_a_ cycle is a strong zeitgeber capable of entraining the LA rhythm in the dromedary camel. However, one can argue that such entrainment of LA rhythms is not specific to camels. Across the literature, several studies have emphasized the effect of the T_a_ cycle on general rest-activity rhythm in various animals, especially non-mammalian vertebrates^42^. Whereas in mammals, only partial entrainment of LA rhythm has been reported in some species, including squirrel monkeys^43^ marmosets^44^, palm squirrels^45^ and mice^46^. Regarding these results, it is quite difficult to distinguish a real entrainment of the central circadian clock from a masking effect on the LA rhythm^46,47^. In the camel, we have previously shown that the circadian clock is synchronized by T_a_ cycles since two robust outputs of the clock, T_b_ and Mel rhythms, are entrained by T_a_ cycles^14^. The observed entrainment of LA rhythm in the present study corroborates these results and confirms the entrainment of the camel SCN by this non-photic cue.

Under heat stress and dehydration, the camel displays an adaptive heterothermy consisting of a switch from a perfect endothermy state to an ectothermy state^12,13^. Indeed, a fully hydrated camel is a perfect endothermic-homeotherm species with constant body temperature not exceeding a daily variation of 2 °C^12,48^. However, when dehydrated and subjected to excessive heat load of the desert, the camel becomes heterothermic^12,49^ functioning like ectothermic-poikilotherms (reptiles, amphibians) with T_b_ passively following the T_a_ cycle. Thus, during the day camels store the heat, but during the night when the thermal gradient becomes negative (T_a_ lower than T_b_), heat is dissipated passively by convection and conduction^50^. Heterothermic camels display daily variations in T_b_ that are life threatening for other mammals, with morning records about 34 °C and evening values of 42 °C^12,13^. This phenomenon is one of the most fascinating adaptive processes to cope with the extreme conditions of the desert. Indeed, adaptive heterothermy allows water economy by preventing the use of evaporative cooling mechanisms that are water consuming. It was reported that this specific thermoregulation state allows a dromedary of 600 kg of body weight to save up to 6 L of water/day (for review see^51^).

The particular T_a_ synchronization of the circadian system in the camel may be related to its specific thermoregulatory system and adaptation to the desert habitat. Possible circadian entrainment by T_a_ depends on the type of thermoregulation displayed by a species. This classifies animals into two categories: ectothermic-poikilotherms in which temperature cycle is a strong zeitgeber capable of synchronizing the central circadian clock, and endothermics-homeothermics for whom T_a_ has a weak effect on the circadian system (For review see Ref^42^). In this general rule, a third type of species is added, the heterothermic species for whom T_a_ has similar effect on the circadian system as in ectothermic-poikilotherm. In fact, it seems that entrainment by T_a_ cycle require a specific sensitivity to T_a_ changes^52–54^, present in ectothermic-poikilothermic and heterothermic species, such as dromedary camels. Recently, heterothermy has also been reported to occur, to some extent, in other desert ungulates like oryx and goat^55,56^. This could explain why the circadian system of the desert black Moroccan goat^15^, displays similar entrainment of T_a_ as is seen in the camel. Accordingly, three outputs of the goat’s central circadian clock, namely the T_b_, LA and Mel rhythms are entrained by T_a_ cycle.

Adaptation to the desert for large mammals like camels, that cannot burrow to avoid heat stress as can small animals, would require coping with the T_a_ cycle by using several strategies. In addition to heterothermy that allows specific thermoregulation and economizing of water, this species could use T_a_ to modulate its circadian physiology. Camels seem to be able to shift the timing of their daily activities depending on the T_a_ cycle. This mechanism is employed by some desert ungulates to reduce heat loads and minimize water loss during the hottest season^55^. It was reported that during the winter season, dromedaries graze during the day and rest at night; while during hot seasons and under solar radiation stress, camels seek shade and become inactive^57^. Such temporal niche switching of activity was demonstrated and intensively investigated in the Arabian oryx, desert bighorn sheep, desert mule deer and other desert mammals^55,58–60^. During the hot season, the circadian rhythm of activity in the Arabian oryx switches from diurnal to crepuscular or even nocturnal. This specific day-time inactivity was demonstrated to be a NREM sleep^61^.

Daily changes in environmental cues, specifically the LD cycle, are commonly used as a predictive and stable external factor for the precise measurement of time throughout the year. Hence, it is a reliable cue for mammals to drive and modulate seasonal rhythms such as reproductive behavior, migration, moulting…which thus occur in the optimal season. However, in desert regions changes in photoperiod are less important than at high latitude. Thus, in some regions an annual variation of only 1-h is observed whilst in high latitudes it can reach 14-h. Although desert animals like camels seem able to integrate even low variations in photoperiod^62^, this alone would not be strong enough to drive seasonal functions. In this regard a strong environmental cue such as T_a_ could be important for driving such rhythms. As previously reported, T_a_ in the desert is known to be a dominant environmental cue able to affect different physiological processes and behavior. At a seasonal rhythmicity level, there are two or three examples in which T_a_ has been experimentally demonstrated to drive seasonal rhythms. In *Spermophilus tridecemlineatus* and *Spermophilus lateralis* two squirrel species living in both forested and arid areas of North America, T_a_ was shown to be a strong zeitgeber much more powerful than the photoperiod, able to modulate and shift circannual rhythms like body weight, hibernation and reproductive activity^63–66^. T_a_ seems also to be important for maintaining seasonal rhythms in two examples of small animals, the European hamster and the edible dormouse (*Glis glis)*, in which under constant photoperiodic regimes (LD or LL), the circannual rhythms of testosterone, thyroxine and activity are present under cyclic T_a_ while they disappear when T_a_ was constant^67,68^. Such demonstrations are unique among mammals because to the best of our knowledge there have been no other attempts to highlight similar findings in other species. Such protocols are difficult to conduct, especially on large mammals such as camels and goats since they are time consuming (2–3 years/breeding cycles), costly and technically difficult, as controlling T_a_ stability for years is complicated.

The desert is a distinctive habitat, in which camels and other ungulates have to face the heat and the T_a_ effects by employing reparatory adaptive mechanisms (heterothermy, renal reabsorption…) but also by using their sensitivity to T_a_ to permit anticipatory adaption. With LD cycles, T_a_ in the desert shapes the activity of animals and defines the temporal division of this circadian rhythm for maintaining energy balance and water economy, specifically during the driest and hottest time of the day. This is probably one of the reasons for which species like the camel have to have a circadian system that can be entrained by T_a_ and shows flexibility to tolerate and avoid the unpredictable environmental conditions that can result in heat stress and dehydration.

## Conclusion

To date the T_a_ cycle entrainment ability of the circadian master clock has been described in only two mammalian species, the camel^14^ and recently in the desert goat^15^. The results of the present work show clearly that locomotor activity, another output of the master circadian clock, is also entrained in the camel by the daily T_a_ cycles. This corroborates the fact that T_a_ daily cycle is a strong environmental cue in the desert habitat, capable of synchronizing the central circadian clock of the camel and the goat. Together, these findings suggest that other desert mammalian species would likely be endowed with a specific circadian system which is modulated by the desert T_a_ cycle variations. Further investigations are still needed to elucidate this hypothesis.

## Methods

### Animals

Six non-pregnant adult female camels (6–9 year) originating from southern Morocco (latitude 23° 43′ N, 15° 57′ S) were used for this study. Animals were housed in specific facilities at the Hassan II Agronomy and Veterinary Medicine Institute of Rabat (Latitude: 34° 01′ N, Longitude: 6° 50′ W). They received a compound feed (Maraa for Camelids, Alf Sahel, Morocco) and barley straw ad libitum and had free access to water. All animal procedures adopted in the present study comply with the ARRIVE (Animal Research: Reporting of In Vivo Experiments) guidelines. The study was in agreement with the Hassan II Agronomy and Veterinary Institute of Rabat and Moroccan Ministry of Agriculture recommendations which are in accordance with international ethical standards (European Union Directive 2010/63/EU) legislation and recommendations in the field of chronobiology^69^.

### Experimental design

*Experiment 1* was designed to determine whether the LA rhythm in camel is driven by a circadian clock and also to demonstrate the entrainment of this rhythm by the LD cycle. The light intensity was 500 lx. Camels were housed individually and were able to move freely in a controlled sheltered stable of 40 m^2^. This experiment was carried out on animals one by one. Each individual experiment consisted of four stages totalling 73 successive days.

- *Stage 1 (7 days)* was an adaptive period of camels to the new indoor conditions. An artificial LD cycle of 12L-12D was applied with an uncontrolled T_a_ (18–24 °C) cycle.
- *Stage 2 (26 days)* This step was intended to demonstrate that the LA rhythm is endogenous. Camels were maintained under constant conditions of total darkness (DD) and constant ambient temperature (CT_a_) of 23.0 ± 0.58 °C to prevent any environmental cyclicity that could provide temporal cues.
- *Stage 3 (22 days)* The CT_a_ conditions (23.0 ± 0.7 °C) were maintained and an LD cycle of 12L: 12D was applied with lights switched on at 07:00 AM.
- *Stage 4 (18 days)* The same conditions as stage 3 were maintained; however, a phase delay of + 6 h was applied to the LD cycle; lights were switched on at 01:00 PM.

*Experiment 2* was conducted to verify if T_a_ cycles are able to entrain LA rhythms in the camel. Similar to the previous experiment (experiment 1), camels were housed individually and allowed to move freely in the same controlled sheltered stable of 40 m^2^. This experiment was also conducted on each individual camel (one by one). One individual experiment lasted 61 successive days and consisted of the following stages:

- *Stage 1 (5 days)* was designed to adapt camels to indoor conditions before starting the following stages. An artificial LD cycle of 12L:12D (light intensity of 500 lx) and a CT_a_ of 18.5–25 °C were applied.
- *Stage 2 (20 days)* As in stage 2 of experiment 1, camels were placed under constant conditions with DD and a CT_a_ of 23.0 ± 0.5 °C.
- *Stage 3 (15 days)* DD conditions were maintained and a T_a_ cycle (NRT: non reversed temperature) was applied with a warmer period (32.5 °C for about 10 h) during the subjective day and the cooler period (16 °C for almost 9 h) during the subjective night. The ascending and descending phases of the T_a_ cycle in which temperatures gradually and respectively increased and decreased were about 2h30min each.
- *Stage 4 (21 days)* DD conditions were kept while the T_a_ cycle was reversed (RT), with a 12-h phase advance to obtain the warmer period (31.9 °C for about 10 h) of the cycle during the subjective night and the cooler period (16.2 °C for almost 10 h) during the subjective day. The ascending and descending phases of T_a_ cycle were about 2 h each.

### Locomotor activity recording

The rhythm of LA was recorded individually in camels using a validated video-locomotion scoring technique^15^. The recorded video sequences were visually analyzed by two well-trained evaluators who assigned 6 scores to each activity state following a predefined scale where:

- *Score 0* correspond to a camel in a sitting position and not moving.
- *Score 1* is a sitting position with slight movements.
- *Score 2* represents a camel in standing position but not moving.
- *Score 3* is a standing camel just moving its limbs with no real locomotion.
- *Score 4* corresponds to a camel walking with exploration of the surrounding area; and
- *Score 5* is assigned to a camel with intense locomotor activity or in an agitated state.

As previously reported^15^, a time set of 5 min was chosen to record LA rhythm in dromedary camels.

### Data analysis

The daily profiles of LA were plotted using Sigma-Plot software (Sigmaplot v12.0, Systat, Chicago, IL). The actograms were plotted using the software Actogram Plotter (Refinetti R, Circadian Rhythm Laboratory, University of South Carolina, https://www.circadian.org/softwar.html).

The LA rhythm parameters, including period, acrophase, mesor, amplitude and robustness, were calculated using nonlinear least squares method with the help of the following software: Cosinor, Acro and LSP software (Refinetti R, Circadian Rhythm Laboratory, University of South Carolina, https://www.circadian.org/softwar.html). This Cosinor analysis was carried out according to the equation:

$$f \, = \, y0 \, + \, a \, * \, \cos \, [2\pi * \, (t \, - \varphi )/t] $$

where *f* is LA at time t, *y0* is the mesor, *a* is the amplitude, *φ* is the acrophase and *τ* is the period. For each parameter, a 95% probability confidence interval is given. Likewise, the degree of significance of the regression was calculated.

Activity onsets and offsets were also determined. The onset represents the phase angle difference in minutes between the time of the beginning of the activity and the time of the cold-hot transition, i.e., time point at which 50% of the ascending phase of T_a_ cycle was reached. While the offset is the phase-angle difference in minutes between the time of the end of activity and the time of the hot–cold transition, i.e., time at half of the T_a_ descending phase.

A one-way ANOVA analysis followed by the Holm-Sidak post-hoc test was used for the statistical comparison of LA rhythm parameters between the different stages of experiments 1 and 2 and also to test the equality of means of the daily activity onsets and offsets around the daily T_a_ transitions of NRT (stage 3) and RT (stage 4) cycles of experiment 2. *P* ≤ 0.05 was considered statistically significant in all statistical tests.

## Supplementary information

Supplementary Information.
