# Supplementary material for: Entrainment of circadian rhythms of locomotor activity by ambient temperature cycles in the dromedary camel
Source: Sci Rep. 2020 Nov 11;10:19515. doi: 10.1038/s41598-020-76535-y (PMC7658228; doi:10.1038/s41598-020-76535-y)
Supplement: Supplementary file 1 — Supplementary Information. [file 41598_2020_76535_MOESM1_ESM.pdf]

Entrainment of circadian rhythms of locomotor activity by ambient temperature cycles in the dromedary camel.

Hicham Farsi[1], Mohamed R. Achaâban[1], Mohammed Piro[2], Béatrice Bothorel[3], Mohammed Ouassat[1], Etienne Challet[3], Paul Pévet[3] and Khalid El Allali[1*]

[1]Comparative Anatomy Unit, Department of Biological and Pharmacological Veterinary Sciences, Hassan II Agronomy and Veterinary Medicine Institute, Rabat, Morocco

[2]Medicine and Surgical Unit of domestic animals, Department of Medicine, Surgery and reproduction, Hassan II Agronomy and Veterinary Medicine Institute, Rabat, Morocco

[3]Institute of Cellular and Integrative Neurosciences, CNRS and University of Strasbourg, Strasbourg, France

Corresponding author

* Khalid El Allali, Comparative Anatomy Unit, Department of Veterinary Biological and Pharmaceutical Sciences, Hassan II Agronomy and Veterinary Medicine Institute, BP: 6202, 10101 Rabat-Instituts, Rabat, Morocco.

Email: k.elallali@iav.ac.ma & khalid_elallali@yahoo.fr

Figure S1: Double plotted actogram of locomotor activity rhythm in four representative dromedary camels during 61 days of experiment 2

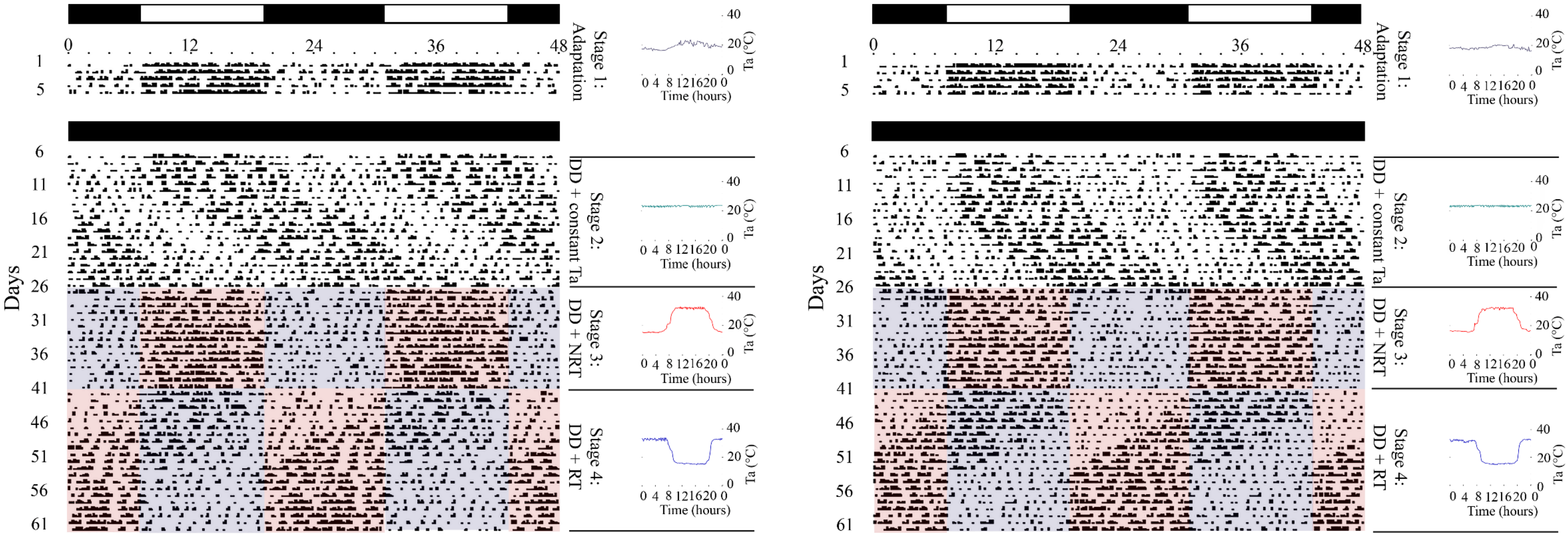

Double plotted actogram of locomotor activity rhythm in camel 3 & 4 during 61 days of experiment 2. Each line corresponds to 24 h of activity starting at 00:00 h and ending at 24:00 h. Black dots and lines denote the existence of a locomotor activity rhythm: scores of 1-5. On-line vacuum corresponds to the absence of activity represented by score 0. The upper black and white bars represent the durations of the LD cycle phases (stage 1); while the long black bar denotes constant darkness (DD) (Stages 2-4). The different T_a cycle regimes of stages 1 to 4 are presented at the right side as 24 h daily cycle. Warmer and cooler periods of T_a cycles are respectively shown within actograms as red and blue areas.

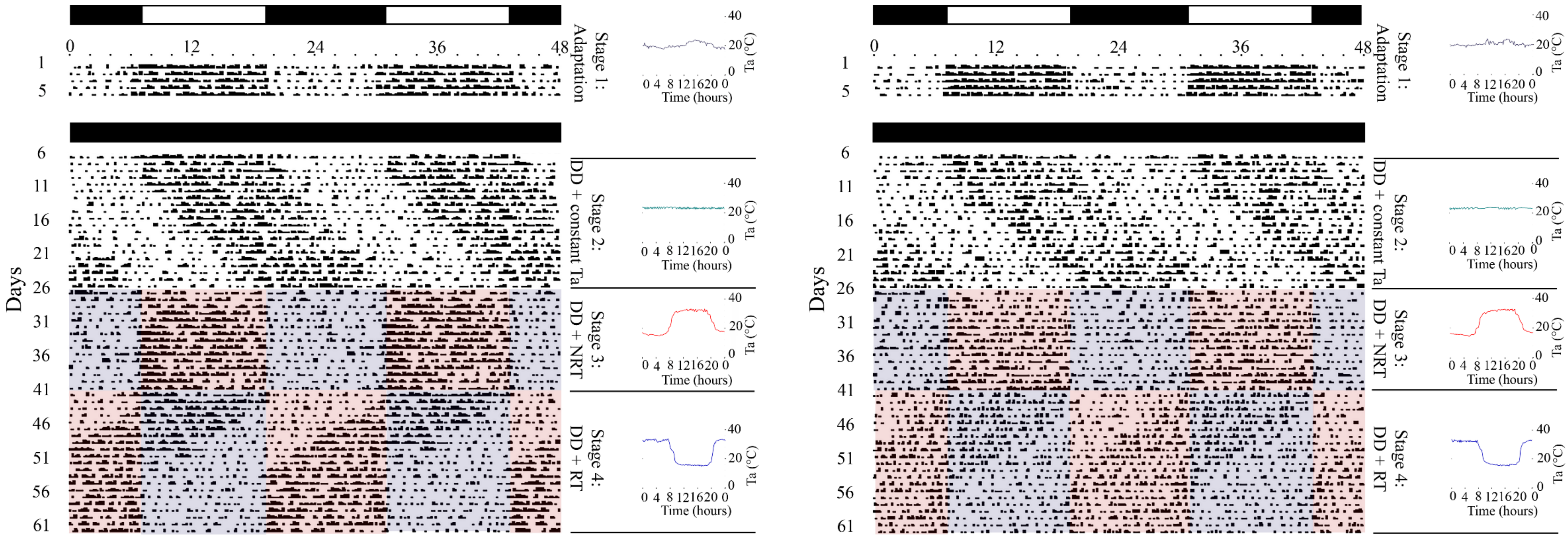

Double plotted actogram of locomotor activity rhythm in camel 5 & 6 during 61 days of experiment 2. Each line corresponds to 24 h of activity starting at 00:00 h and ending at 24:00 h. Black dots and lines denote the existence of a locomotor activity rhythm: scores of 1-5. On-line vacuum corresponds to the absence of activity represented by score 0. The upper black and white bars represent the durations of the LD cycle phases (stage 1); while the long black bar denotes constant darkness (DD) (Stages 2-4). The different T_a cycle regimes of stages 1 to 4 are presented at the right side as 24 h daily cycle. Warmer and cooler periods of T_a cycles are respectively shown within actograms as red and blue areas.
